# Phlebotomy in Excelsis

**Published:** 1888-07-14

**Authors:** W. Curran


### Phlebotomy in Excelsis.

By Dr. W. Curran.

Whoever will take the trouble of looking through that popular little book yclept "Selections from Physicians' Prescriptions," by the late Dr. Paris, will find numerous references to, as well as equally numerous directions for, "the letting of blood" in his fellow-man. Whether this practice obtained in the Stone Age we are now unable to say; but some of the weapons or implements of that remote period are so fashioned as almost to imply its existence, and savage man has ever retained, or at least had a liking for it. Thus the Dacotas are, as the Abbé Domenech tells us in "The Great Deserts of North America," "very fond of scarifications of various kinds, while the Natchez bleed successfully"; and, describing the Patagonians, Captain Masters says ("At Home with the Patagonians," p. 183) that "they practise blood-letting, not only on the sick, but, like our ancestors, at regular seasons have themselves blooded, believing it to be beneficial. Casimir declares, he adds, that the superior health of the Tehuelches, compared with that of the colonists or Christians, was attributable to this practice"; and the writings of other travellers or explorers are equally explicit in their ascription of this measure to other savage or semi-civilized tribes and peoples.

Though there is no mention of the matter—so far as I can discover—in the writings of Homer or Hesiod, while the articles discovered at Mycenae by Dr. Schliemann do not bear any conclusive evidence in its favour, yet is its antiquity very great; so great is it indeed that it is lost in the mist of ages; and I have not now access to any of those seventeen books that (as old Burton tells us, "The Anatomy of Melancholy,' p. 448), "Horatius Eugenius, a physician of Padua, hath lately writ about it." This being so, while the question is more a matter of antiquarian curiosity than of practical interest, we may turn to other better-known phases of the case, and see what of value or certainty, if any, these teach us.

To show the continuity and prevalence of this practice in ancient times, I may here mention that Celsus—who wrote before the birth of Christ—gives minute directions as to the diseases in which it (blood-letting) should be resorted to; and that Juvenal—who was born about the year of Christ 38—advises as follows respecting it (see Gifford's translation):—

> But what! Ursidius hopes a mate to gain,
> Frugal and chaste and of the good old strain.
> Alas, he's frantic! Ope a vein with speed,
> And bleed him copiously, good doctor, bleed.

There are also numerous allusions to, or directions about, this practice in the "Scola Salernitana," pp. 424—7, etc.

In the very able and interesting address in medicine with which Dr. Gairdner, of Glasgow, entertained his confrères at Dublin in August last, he says, *inter alia*, "I must ask you to take it as the matured conviction of one who has given some attention to the subject, that the genuine Hippocratic tradition was not nearly so extreme in respect of blood-letting as that of the mediæval"; and that, too, is the outcome of my own reading or research on the subject. Thus we learn from various sources that this practice of blood-letting was so much in vogue at the beginning of the sixth century that the reigning emperor, Theodoric the Great, issued a law which imposed a fine of 100 solidi (about fifty guineas of our money) on every physician or surgeon who had harmed a noble by venesection. If the noble died as the result of this practice, his relatives were (*Lancet*, March 8th, 1888) by the same law empowered to lay hands on the practitioner, and mete out to him such punishment as they pleased; and we know from one of the many authorities quoted by old Burton that "it was an ordinary thing of old, in Galen's time, to take at once from (melancholic) men six pounds of blood." We also know that "the great zoologist Buffon defended the practice, centuries afterwards, on the ground that a sick ourang-outang made signs with its arms that it wanted a vein opened"; and Alexander Trallianus expressly tells us that "to open the veins of the forehead, nose, and ears simultaneously, and especially so in the head, is good."

We need not here discuss the directions given by Dr. Paris for the performance of this operation, as it is now so rarely resorted to as to be all but extinct among us; but a process that has been employed by such men as Bailey, Copland, and Marshall Hall in our own days should not be lightly discarded; and it is well known that many of our older practitioners retain still a sneaking liking for it. Be that, however, as it may, and ignoring for the present Pliny's hints as to the hippopotamus and his performances in connexion with it, we may allude to the practice of the famous James Gregory, who was so fond of it that he held that "it is often necessary to bleed twice, and sometimes three or four times, in the day" during the progress of a pneumonia; as well as to that of the more renowned Dr. Sangrado, who ordered a surgeon "to take six good porringers of blood" from one of his patients, 'as a first effort,' in order to supply the want of perspiration." He further laid it down as a principle about which there ought to be no doubt, that "a patient cannot be blooded too much"; and this, too, was at one time the belief of hundreds of other Sangrados in this and other more or less enlightened countries.

We have only to look, for proof in point, into any of the older or more popular classics of our art. *Si quaeris monumentum circumspice*; and the curious reader will find ample evidence of this in the recent writings of Sir Charles Cameron, of Dublin, as well as in the late Mr. South's curious "Memorials of the Craft of Surgery in England." It is mentioned by Fell in his "Life of Dr. Hammond," p. 365 (who suffered repeatedly during his last illness from epistaxis), that so violent or uncontrollable was this flux that "the opening of a vein, first in the arm and after in the foot," had to be resorted to. "At last, the fountain being exhausted, the torrent ceased its course." The poor sufferer's life ceased too soon afterwards, and the following lines from the quaint old ballad of "Robin Hood's Death and Burial" are so suggestive of the popular feelings and practices of the time that I am induced to reproduce them in full here.

Describing his arrival at "fair Kirkley Hall," and his reception thereat, the writer says:

V.

"Will you please to sit down, cousin Robin," she said,
"And drink some beer with me?"
"No, I will neither eat nor drink
Till I am blooded by thee."

VI.

"Well, I have a room, cousin Robin," she said,
"Which you did never see;
And if you please to walk therein,
You blooded by me shall be."

VII.

She took him by the lily-white hand,
And led him to a private room,
And there she blooded bold Robin Hood
Whilst one drop of blood would run.

VIII.

She blooded him in the vein of the arm,
And locked him up in the room;
There did he bleed all the livelong day,
Until the next day at noon.

*Apropos* of this strange rage for bleeding, the following passage from "Robinson Crusoe" may prove interesting. Alluding to his escape from drowning, as well as to the sensations that drowning men often feel, he (R.) says that: "I believe it is impossible to express to the life what the ecstacies and transports of the soul are when it is saved, as I may say, out of the grave, and I did not wonder now at the custom, viz., that when a malefactor, who has the halter about his neck, and just going to be turned off, and has a reprieve brought to him; I say, I do not wonder that they bring a surgeon with it, to let him blood that very moment they tell him of it, that the surprise may not drive the animal spirits from the heart and overwhelm him." This is curious if true, but it is well known that Defoe wrote most of his romances with a purpose, or from life, and it may be so in this instance. And we need not stop to inquire about the result of this strange bleeding. All we may here say anent it is that such a process would now in all probability entail a "crowner's 'quest," and possibly also—an enforced visit to Newgate. So keen (says Mr. South) was at one time the competition for this kind of practice between those barber-surgeons who were the predecessors of our own Brodies and Lawrences, that they actually vied with one another in displays of their talents. They exhibited the blood of their victims, in various stages of solution or coagulation, in porringers, in open day and open spaces, outside their surgeries or shop windows; and the curious searcher after surgical curiosities will find further evidence of this now happily obsolete practice in the drawings of Hogarth, Rowlandson, or others of the caricaturists of those days. He will also find it in another form in Rabelais and Molière, and as to our own Sternes and Smolletts, their sentiments about it are so well known as not to need or call for recapitulation here.

Coming nearer home, there is evidence to show that the practice is not dead, but merely sleepeth among ourselves. Describing his own habits, the late eccentric Mr. Waterton mentions in a letter quoted in "Notes and Queries" for November 15th, 1879, p. 385, that "I have blooded myself more than one hundred and sixty times with my own hand, since I was twenty-four years old." So also we learn from the "Greville Memoirs," vol. i., pp. 23, 24, that "the new king has been desperately ill. He had a bad cold at Brighton, for which he lost eighty ounces of blood; yet he afterwards had a severe oppression, amounting almost to suffocation, in his chest. . . . . Bloomfield sent for Tierney, who took fifty ounces from him. . . . . On Wednesday he lost twenty ounces more. . . . . Tierney certainly saved his life, for he must have died if he had not been blooded," And though modern diagnosis or pathology will scarcely endorse this very confident dictum, they yet do not deem it to be either incredible or absurd. A lusty plethoric man like this "new king" might have gone farther and fared worse. That many precious lives have been sacrificed to or by the votaries of this fashion in times past, there can be no doubt, and the story of Count Cavour (who was said to have been done to death in this way) is still familiar to all of us. So is that, though in a lesser degree, of the unfortunate Elizabeth of France, who appears to have been "disposed of" after an even more bloody encounter of the same kind. Speaking of her, Challoner, the English Ambassador, says (Froude's "History of England," vol. viii. p. 45) that "they bled her in both arms; they bled her in both feet, and when spasms and paroxysms came on they cupped her, and then gave her up and left her to die." One almost regrets, in face of such barbarity, that the statute of Theodoric did not remain in force.

